# IP-10 acts early in CV-A16 infection to induce BBB destruction and promote virus entry into the CNS by increasing TNF-α expression

**DOI:** 10.3389/fimmu.2024.1374447

**Published:** 2024-11-04

**Authors:** Yajie Hu, Yunguang Hu, Anguo Yin, Yaming Lv, Jiang Li, Jingyuan Fan, Baojiang Qian, Jie Song, Yunhui Zhang

**Affiliations:** ^1^ Department of Pulmonary and Critical Care Medicine, The First People’s Hospital of Yunnan Province, Kunming, China; ^2^ Department of Pulmonary and Critical Care Medicine, The Affiliated Hospital of Kunming University of Science and Technology, Kunming, Yunnan, China; ^3^ National and Local Engineering Center for Infectious Biological Products, Institute of Medical Biology, Chinese Academy of Medical Science and Peking Union Medical College, Kunming, China

**Keywords:** hand foot and mouth disease (HFMD), coxsackievirus A16 (CV-A16), IFN-γ-inducible protein-10 (IP-10), blood-brain barrier (BBB), neuroinflammation

## Abstract

The mechanisms underlying pathological changes in the central nervous system (CNS) following Coxsackievirus A16 (CV-A16) infection have not yet been elucidated. IFN-γ-inducible protein-10 (IP-10) is often used as a predictive factor to monitor early virus infection. It has also been reported that IP-10 plays a pivotal role in neuroinflammation. In this study, we aimed to explore the role of IP-10 in the neuropathogenesis of CV-A16 infection. We observed that the level of IP-10, as well as the TLR3-TRIF-TRAF3-TBK1-NF-κB and RIG-I/MDA5-MAVS-TRAFS-TBK1-NF-κB pathways, which are the upstream of IP-10, were significantly elevated during the course of CV-A16 infection. This increase was accompanied by an increase in a series of inflammatory cytokines at different time-points during CV-A16 infection. To determine whether IP-10 influences BBB integrity, we examined junctional complexes. Our results revealed that the expression levels of Claudin5, Occludin, ZO-1 and VE-Cadherin were notably decreased in CV-A16-infected HUVECs, but these indicators were restored in CV-A16-infected HUVECs with Eldelumab treatment. Nevertheless, IP-10 is only a chemokine that primarily traffics CXCR3-positive immune cells to inflammatory sites or promotes the production of inflammatory cytokines. Therefore, the interactions between IP-10 and inflammatory cytokines were evaluated. Our data revealed that IP-10 mediated the production of TNF-α, which was also observed to change the junctional complexes. Moreover, in a suckling mouse model, IP-10 and TNF-α treatments exacerbated clinical symptoms, mortality and pathological changes in the brain of CV-A16-infected mice, but Anti-IP-10 and Anti-TNF-α treatments alleviated these changes. Our data also revealed that IP-10 may be detected early in CV-A16 infection, whereas TNF-α was detected late in CV-A16 infection, and the production of TNF-α was also found to be positively correlated with IP-10. In addition, IP-10 and TNF-α were observed to reduce junctional complexes and enhance virus entry into the CNS. Taken together, this study provides the first evidence that CV-A16 activates the IP-10/TNF-α regulatory axis to cause BBB damage and accelerate the formation of neuroinflammation in infected hosts, which not only provides a new understanding of the neuropathogenesis caused by CV-A16, but also offers a promising target for the development of CV-A16 antiviral drugs.

## Introduction

Hand, foot and mouth disease (HFMD) is a viral illness commonly observed in children less than 5 years of age and is caused primarily by enterovirus 71 (EV-A71) and coxsackievirus A16 (CV-A16) (1). The usual presentations are fever, oral ulcerations, and papules on the palms of the hands and the soles of the feet, with a recovery time of less than 1 week; however, a minority of severe cases may present with clinical complications with neurological symptoms, such as aseptic meningitis, brainstem encephalitis and acute flaccid paralysis, etc (1, 2). Previous animal model studies and vaccine development have been focused more on EV-A71 because of the higher prevalence of severe or even fatal central nervous system (CNS) complications (2), but recent studies have demonstrated that although the vast majority of CV-A16 infections are generally considered benign and self-limiting, they can similarly cause severe neurological complications (3). To date, inactivated monovalent EV-A71 vaccines have been developed and have shown a highly effective protection rate for EV-A71-associated HFMD (4). Unfortunately, these vaccines cannot induce a cross-neutralization response against CV-A16 or other serological viruses (4, 5). Epidemiological investigations have shown that since EV-A71 vaccines were first introduced in China, the HFMD pathogen spectrum and epidemiological trend have significantly changed (6, 7). The proportion of EV-A71 has decreased and the proportion of CV-A16 has increased; thus CV-A16 has become a severe threat to human health. CNS lesions are fatal changes induced by CV-A16 infection (3). A previous study had revealed that the distribution of inflammatory lesions in the pons and cerebellum of CV-A16-associated patients may be similar to that of patients with EV-A71 encephalomyelitis (4, 8), but little is known about the CNS pathology in CV-A16 infection. Research in our lab has focused on the mechanism by which enterovirus infection induces inflammatory damage in the CNS. For example, it has been reported that CV-A16 may penetrate the BBB and then enter the CNS by downregulating miR-1303, which disrupts junctional complexes by directly regulating MMP9 and ultimately causing pathological CNS changes (9). CV-A10 also may destroy the junction complexes in the BBB by increasing the level of miR-143-3p, leading to destruction of BBB integrity (10). Moreover, other studies have demonstrated that EV-A71 can disrupt the BBB via different routes, such as increasing the expression of the virus receptor vimentin (11) or releasing the inflammatory mediator HMGB1 (12) in the BBB. These results suggest that enterovirus destruction of the BBB is the mechanism underlying of its entry into the CNS. Therefore, finding key indicator molecules much earlier in the clinic before enteroviruses destroy the BBB is a priority to avoid enterovirus-induced severe illness. Exploring the CNS pathogenesis of CV-A16 infection can provide not only a new biological target for early clinical diagnosis and treatment, but also a new theoretical basis for the development of a globally representative multivalent HFMD vaccine.

Numerous studies have shown that cytokines and chemokines play an important roles in the occurrence and development of infectious disease (13). Moreover, systemic inflammatory cytokine storms are characteristic of critical severe HFMD patients and are closely associated with the pathogenesis of severe CNS complications (14). An autopsy report of a child with pulmonary edema due to EV-A71 infection revealed extensive infiltration of inflammatory cells in the brain stem and spinal cord (15). Dysregulated inflammatory cytokines and chemokines, including interleukin (IL)-1β, IL-6, IL-8, IL-17F, IL-18, tumor necrosis factor (TNF)-α, interferon (IFN)-γ, IFN-γ-inducible protein-10 (IP-10), monocyte chemoattractant protein (MCP)-1, granulocyte colony-stimulating factor (G-CSF), and high mobility group box 1 (HMGB1) have been identified in the serum or cerebrospinal fluid (CSF) of severe HFMD patients (14, 16). Therefore, changes in inflammatory factors are highly important during enterovirus infection. Among the inflammatory factors mentioned above, we focused on IP-10, also known as C-X-C motif ligand (CXCL) 10, which is widely produced by various cell types and performs its antiviral immune function by acting as a chemoattractant for leukocytes (17). Currently, several clinical studies have demonstrated that the baseline level of IP-10 is a crucial predictive factor in the course of viral infection (18). For example, blood serum IP-10 levels have emerged as a prognostic indicator for patients with mild, moderate and severe COVID-19 infection (19). IP-10 plasma levels are elevated in most human immunodeficiency virus (HIV)-infected individuals, and IP-10 is the only cytokine that is consistently associated with HIV disease progression during the acute HIV infection period (20). Chronic obstructive pulmonary disease patients with human rhinovirus (HRV) infection were found to have high serum concentrations of IP-10, which is considered as a marker from baseline to exacerbation (21). Moreover, accumulating evidence has also indicated that the concentrations of IP-10 are significantly high in EV-A71-related patients, especially in children with severe illness caused by EV-A71 (22, 23). Therefore, high levels of IP-10 have been observed in several viral diseases, but its mechanism in the pathogenesis of viral diseases remains unknown. Additionally, the roles of IP-10 in several CNS diseases, such as tickborne encephalitis, Alzheimer’s disease, multiple sclerosis, and spinal cord injury, have been reported in the literature (24, 25). These studies of CNS diseases reported that both of serum and CSF IP-10 concentrations are significantly elevated; moreover, the concentration gradients of IP-10 between the CSF and the serum may recruit T lymphocytes to the CSF, which is related to neuroinflammation (24). Therefore, on the basis of the above research, the purpose of this study was to explore the neuroinflammatory mechanism of IP-10 during CV-A16 infection.

In this study, we used cellular and animal models to explore the role of IP-10 in BBB disruption during CV-A16 infection. The results showed that after CV-A16 infection, IP-10 can be activated through the PRR-mediated pathways, and then IP-10 induced TNF-α production leads to BBB permeability alteration via destroying the intercellular connection at the BBB; thereby these findings suggest that the IP-10/TNF-α cytokine axis can be considered as a new marker for early diagnosis in CV-A16-infected HFMD patients, as well as a potential target for developing novel therapies for CV-A16-infected HFMD patients.

## Materials and methods

### Cells and viruses

Human umbilical vein endothelial cells (HUVECs) were obtained from the American Type Culture Collection and cultured in Dulbecco’s modified Eagle’s medium (DMEM; Corning, USA) supplemented with 10% fetal bovine serum (FBS; Gibco, USA) and 1% antibiotics (penicillin-streptomycin) in a humidified atmosphere containing 5% CO_2_ at 37°C.

The CV-A16-G20 strain (subgenotype B, GenBank: JN590244.1), which was isolated from an HFMD patient in Guangxi, China in 2010, was used for these experiments. Monolayers of HUVECs were plated into 6-well plates overnight and then infected with CV-A16 at a multiplicity of infection (MOI) of 0.1. After 2 h of adsorption, the virus inoculum was removed, and the cells were maintained in DMEM supplemented with 2% FBS at 37°C. The cells and supernatants were harvested at the indicated time points.

Next, to investigate the effect of NF-κB on the production of IP-10 and the effect of IP-10 on BBB integrity, HUVECs were transfected with NF-κB-knockdown or NF-κB-overexpression plasmids and treated with Eldelumab (a monoclonal antibody against IP-10), respectively.

### Quantitative reverse transcription-polymerase chain reaction for *IP-10* expression

For the quantification of *IP-10* mRNA, the following primers were used: (forward: 5’-AGTTAGCAAGGAAAGGTCT-3’, reverse: 5’-ACATTATAGTGCCAGGT-3’) were used in this study. Total RNA was extracted from cells via TRIzol reagent (TIANGEN, China) according to the manufacturer’s protocol. The quality and concentration of RNA were measured by the ratio of absorbance at 260/280 nm via a NanoDrop 2000 spectrophotometer (Thermo Fisher Scientific, USA). The RNA was subjected to cDNA synthesis with a PrimeScript™ RT reagent Kit (TAKARA, Japan), followed by qRT-PCR with SYBR Premix Ex Taq (TAKARA, Japan) on an ABI7500 real-time PCR system (Applied Biosystems, USA). The data were normalized to the level of GAPDH expression in each individual sample, and the 2^−ΔΔCt^ method was used to calculate relative expression changes.

### Western blotting analysis

WB analysis was performed as described below. Briefly, total proteins were extracted from cell lines or tissues with cell lysis buffer containing 1 mM PMSF (Beyotime, China) at 4°C for 30 min, followed by centrifugation at 12,000 rpm for 10 min to collect the supernatant. The protein concentrations of the lysates were quantified with a BCA protein assay kit (Beyotime, China). Then, the lysates were resuspended in 2 × loading buffer and boiled at 100°C in boiling water for 10 min. Equal protein samples were electrophoretically separated by sodium dodecyl sulfate polyacrylamide gel electrophoresis (SDS-PAGE) at a concentration of 10% and transferred to transferred onto a polyvinylidene difluoride (PVDF) membranes (Millipore, USA). The membranes blocked with 5% skim milk for 2 h at room temperature, reacted with the indicated primary antibodies, including IP-10 (1:1,000 dilution; ABclonal, China; Cat NO. A21986), TLR3 (1:1,000 dilution; ABclonal, China; Cat NO. A24885), TRIF (1:1,500 dilution; Affinity, USA; Cat NO. DF6289), RIG-I (1:1,000 dilution; ABclonal, China; Cat NO. A23179), MDA5 (1:1,000 dilution; ABclonal, China; Cat NO. A2419), MAVS (1:1,500 dilution; Affinity, USA; Cat NO. DF12211), TRAF3 (1:1,500 dilution; Affinity, USA; Cat NO. AF5380), TBK1 (1:1,500 dilution; Affinity, USA; Cat NO.DF7026), NF-κB (1:1,000 dilution; Affinity, USA; Cat NO. AF5006), Claudin5 (1:1,500 dilution; Affinity, USA; Cat NO. AF5216), Occludin (1:1,500 dilution; Affinity, USA; Cat NO. DF7504), ZO-1 (1:1,000 dilution; ABclonal, China; Cat NO. A11417), VE-Cadherin (1:1,500 dilution; Affinity, USA; Cat NO. AF6265), VP1 (1:1,000 dilution; Millipore, USA; Lot Cat 3822821) and GAPDH (1:5,000 dilution; Abbinke, China; Cat NO. ABL1025), overnight at 4°C, followed by the corresponding horseradish peroxidase (HRP)-conjugated secondary antibodies (1:1,0000 dilution; Abbinke, China; Cat NO. A21010 and A21020) for 2 h at room temperature. The membranes were washed with 0.1% Tween 20/PBS three times after each step. The immunoreactive bands were visualized using an enhanced chemiluminescence (ECL) system (Biosharp, China).

### Enzyme-linked immunosorbent assay

ELISA was performed to detect the concentrations of IP-10, IL-1β, IL-6 or TNF-α via the double-antibody sandwich method. Commercial kits, namely the Human IP-10 ELISA Kit (Abcam, USA; Cat NO. ab173194), the Human IL-1β ELISA Kit (NeoBioscience, China; Cat NO. EHC002bQT.96), the Human IL-6 ELISA Kit (NeoBioscience, China; Cat NO. EHC007QT.96) and the Human TNF-α ELISA Kit (NeoBioscience, China; Cat NO. EHC103aQT.96), were used following the manufacturers’ instructions. The concentrations of IP-10, IL-1β, IL-6 or TNF-α were calculated according to their standard curves.

### Confocal fluorescence microscopy

HUVECs plated on coverslips in 24-well plates were infected with CV-A16 for 24 h, washed with phosphate-buffered saline (PBS) and fixed with 4% paraformaldehyde (Biosharp, China) for 30 min. The cells were then incubated with 0.1% Triton X-100 for 10 min and blocked with 2% bovine serum albumin (BSA; Biofroxx, China) for 1 h at room temperature. Next, the cells were incubated with primary antibodies at a dilution of 1:200 in PBS, including NF-κB, Claudin5, Occludin, ZO-1 and VE-Cadherin, overnight at 4°C, followed by immobilization with Alexa Fluor^®^ 488-conjugated goat-anti-mouse IgG (1:300 dilution; CST, USA; Cat NO.4408S) and Alexa Fluor^®^ 594-conjugated goat-anti-rabbit IgG (1:300 dilution; CST, USA; Cat NO.8889S) for 2 h in the dark. The cells were washed three times with PBS after each incubation, and the nuclei were stained with 4’,6-diamidino-2-phenylindole (DAPI). Finally, photographs were taken with a laser confocal microscope (Leica, Germany).

### Flow cytometry analysis of inflammatory cytokines

Detection of inflammatory cytokines was performed via flow cytometry. Briefly, 25 μl of sample, 25 μl of trapped microspheres and 25 μl of experimental buffer were mixed and incubated for 2 hours with shaking. Then, 25 μl of SA-PE antibody was added, and the mixture was incubated for 30 min. Finally, after cleaning with 100 μl of washing buffer and centrifuging for 5 min at 1,500 rpm/min, 300 μl of washing buffer was added, and the mixture was analyzed on a flow cytometer. The levels of inflammatory cytokines were calculated using a LEGENDplex v8.0 software.

### Mouse experiments

The animal experiments were conducted according to the protocol (number: DWSP202312004) approved by the Animal Ethics Committee of Institute of the Medical Biology, Chinese Academy of Medical Sciences. All procedures performed in studies involving animals were in accordance with the Guide for the Care and Use of Laboratory Animals.

Pregnant BALB/C mice obtained from the Experimental Animal Center of the Institute of Medical Biology, Chinese Academy of Medical Sciences, were randomly divided into the following 10 groups (N=12): Control, CV-A16, IP-10, Anti-IP-10, IP-10+CV-A16, Anti-IP-10+CV-A16, TNF-α, Anti-TNF-α, TNF-α+CV-A16 and Anti-TNF-α+CV-A16. The groups of suckling mice were treated as follows: mice in the IP-10-related treatment groups were intraperitoneally injected with 50 μl of 100 ng/ml IP-10 recombinant protein (ABclonal, China); mice in the Anti-IP-10-related treatment groups were intraperitoneally injected with 50 μl of 100 ng/ml IP-10 antibody (Abmart, China); mice in the TNF-α-related treatment groups were intraperitoneally injected with 50 μl of 50 ng/ml TNF-α recombinant protein (MCE, USA); and mice in the Anti-TNF-α-related treatment groups were intraperitoneally injected with 50 μl of 5 mg/kg TNF-α recombinant protein (MCE, USA).

Suckling mice were clinically examined daily, including for survival and clinical symptoms. At Day 4 and Day 8, blood samples were collected, and brain tissues were removed immediately after all the animals were sacrificed painlessly. Blood samples were used for the examination of IP-10, IL-1β, IL-6 or TNF-α via ELISA. The brain tissues were stored at -80°C prior to testing the viral dissemination of CV-A16 via qRT-PCR and inflammatory cytokines by flow cytometry. The brain tissues were also collected, fixed with 10% formalin, and then embedded in paraffin before cutting 5 μm coronal sections. The sections were then used for hematoxylin and eosin (H&E) and immunohistochemical (IHC) staining.

### H&E staining

Pathological changes in brain tissues were observed by H&E staining. Deparaffinized slides were stained with hematoxylin for 5 min and eosin for 2 min at 25°C. Then, the stained slides were sealed with a neutral resin and images were acquired using a digital pathology slide scanner (KFBIO, China).

### qRT-PCR for determining the viral load

qRT-PCR was used for the examination of the viral load and was conducted as previously described. In brief, the RNA of mouse brains was extracted with TRIzol reagent (TIANGEN, China) according to the manufacturer’s guidelines. The qRT-PCR process was subsequently performed using a One Step PrimeScript™ RT-PCR kit (TAKARA, Japan) on a GENTIER 96 instrument (TIANLONG, China). Moreover, the standards for CV-A16 at known concentrations were measured concurrently with the test samples. The viral load of each sample was subsequently calculated from the standard curve generated from the CV-A16 standards.

### IHC staining

Paraffin-embedded brain sections (5 μm thick) were deparaffinized and rehydrated. Subsequently, the sections were subjected to antigen retrieval with citrate antigen retrieval solution (Servicebio, China) by microwaving for 10 min and then blocked with 5% BSA in PBS. CV-A16-VP1 protein monoclonal antibody (1:100 dilution) was applied overnight at 4°C. Following rinses three times with PBS, the sections were further incubated with HRP-conjugated secondary antibody (1:800 dilution) at room temperature for 1 h. Finally, diaminobenzidine (DAB; Servicebio, China) was used as a substrate for color development, and the sections were observed with a digital pathology slide scanner (KFBIO, China).

### Statistical analysis

All results are representative of 3 independent experiments, and the data are presented as the means ± standard deviations (SDs) and were analyzed via GraphPad Prism 7.0 software. The statistical significance of differences between two groups was evaluated using Student’s t test, but three groups were analyzed using a one-way ANOVA with Dunnett’s test, and multiple groups were analyzed using a two-way ANOVA with Tukey’s multiple-comparisons test. Differences were considered statistically significant when *P* < 0.05.

## Results

### CV-A16 induces IP-10 mRNA and protein release in a time-dependent fashion

**Figure 1:**
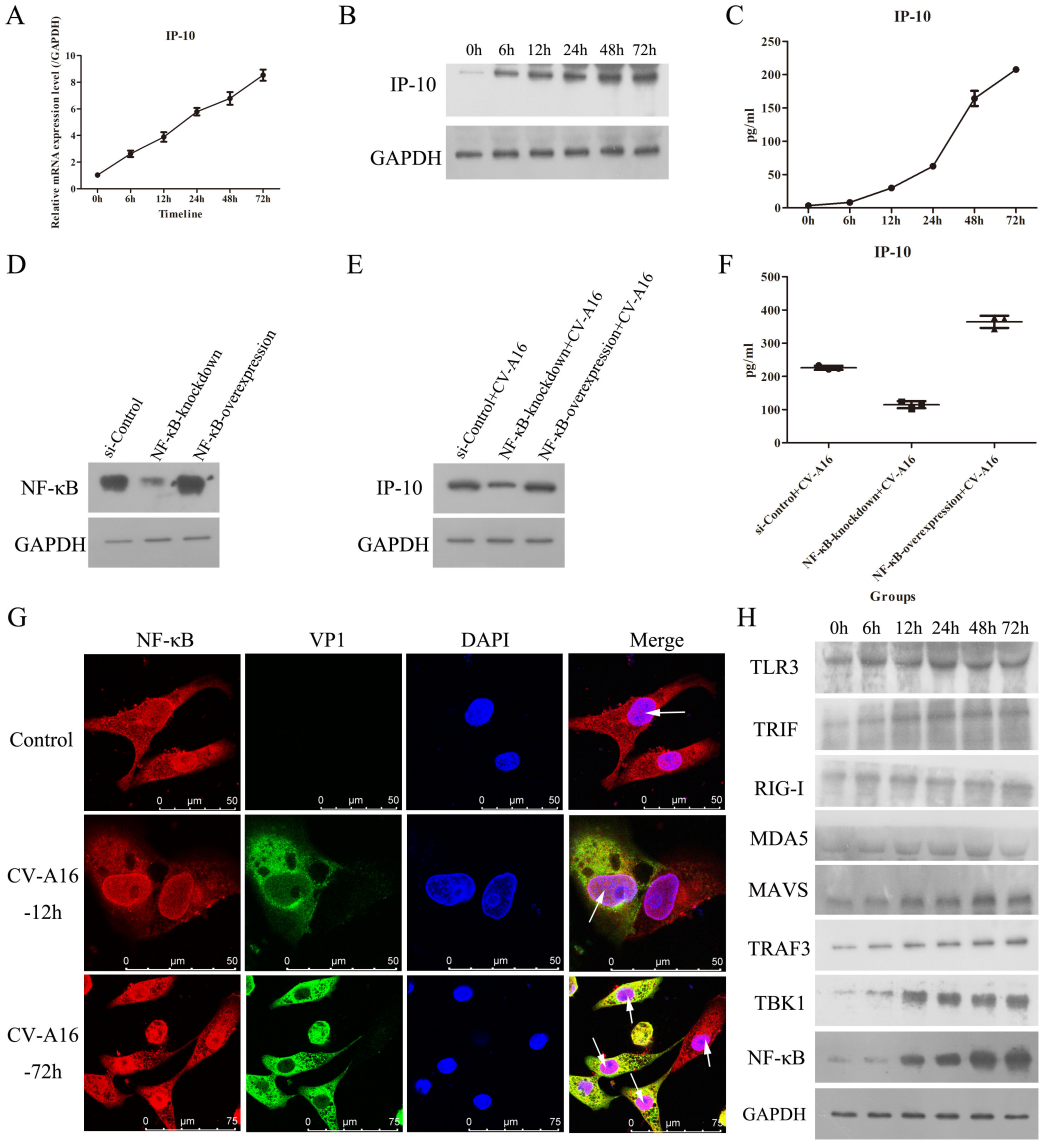
CV-A16 infection up-regulates NF-κB-mediated IP-10 production. **(A)** The mRNA expression of IP-10 was measured via qRT-PCR. **(B)** The protein expression of IP-10 was detected by WB. **(C)** The release concentration of IP-10 was determined by ELISA. **(D)** NF-κB protein expression was examined with WB in NF-κB-knockdown or NF-κB-overexpressing treated cells. **(E)** The protein expression of IP-10 was assessed using WB in NF-κB-knockdown or NF-κB-overexpressing treated cells. **(F)** The concentration of IP-10 released from NF-κB-knockdown or NF-κB-overexpressing cells was monitored via ELISA. **(G)** The fluorescence signals of NF-κB were visualized by confocal immunofluorescence microscopy. **(H)** WB analysis of TLR3-TRIF-TRAF3-TBK1-NF-κB and RIG-I/MDA5-MAVS-TRAFS-TBK1-NF-κB signaling molecules in CV-A16-infected cells.

IP-10 is often up-regulated in the early stage of many viral infections, making IP-10 useful as a marker of early viral infection (18). However, how IP-10 changes during CV-A16 infection is not known. As shown in **Figures 1A, B**, qRT-PCR and WB data revealed that CV-A16 infection of HUVECs caused a significant, time-dependent increases in the mRNA and protein expression of IP-10, respectively. Moreover, the ELISA results also revealed that the cumulative protein level of IP-10 protein in culture supernatants from CV-A16-infected cells clearly increased over time (**Figure 1C**). Thus, these results suggest that CV-A16 infection rapidly promotes IP-10 expression.

### The NF-κB signaling pathway is involved in IP-10 expression

Previous studies have been reported that the IP-10 expression involves the activation of many pathways, mainly including the p38-, JNK-, ERK-, Akt- and NF-κB-mediated pathways (18). Since IP-10 is a proinflammatory factor, we focused on the NF-κB signaling pathway. Moreover, because CV-A16 is an RNA virus, we investigated 2 pattern recognition receptor pathways associated with NF-κB, namely, TLR3-TRIF-TRAF3-TBK1-NF-κB and RIG-I/MDA5-MAVS-TRAFS-TBK1-NF-κB. Therefore, we first investigated the relationship between NF-κB and IP-10. To demonstrate that the activation of NF-κB can promote IP-10 expression, we transfected NF-κB-knockdown and NF-κB-overexpression vectors into cells, and the WB results revealed that plasmid transfection of NF-κB was effective (**Figure 1D**). Then, it was found that NF-κB-knockdown decelerated IP-10 expression, and NF-κB-overexpression accelerated IP-10 expression during CV-A16 infection (**Figures 1E, F**). These data indicate that NF-κB can regulate the expression of IP-10.

Next, IF staining revealed that during CV-A16 infection, NF-κB was markedly increased in the nucleus (**Figure 1G**). Moreover, PPR-related molecules involved upstream of NF-κB were markedly elevated during CV-A16 infection with increasing infection time (**Figure 1H**). These findings suggest that CV-A16 infection may improve the NF-κB signaling pathway by activating innate immunity.

### CV-A16 infection activates the inflammatory response

**Figure 2:**
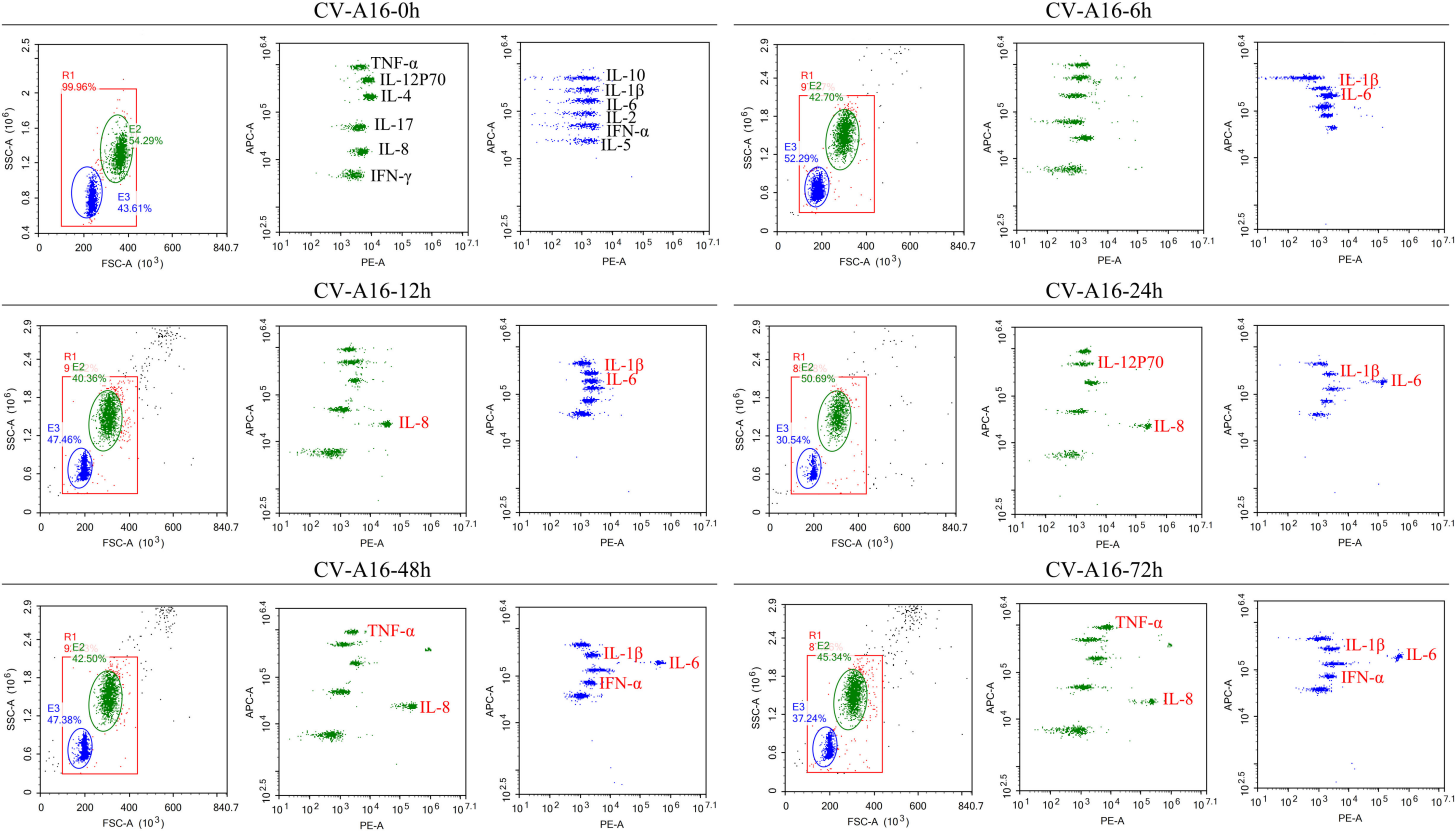
CV-A16 infection promotes the inflammatory response. Flow cytometry was utilized to examine 12 inflammatory cytokines. The inflammatory cytokines in the red font are the cytokines that changed.

NF-κB is a critical regulator of the immediate early pathogen response and plays an essential role in inflammatory responses (26, 27). The above results indicated that NF-κB was activated, so we further explored whether the inflammatory response under the downstream of NF-κB was also activated. It was observed that IL-1β and IL-6 were the first to be upregulated at 6 hours after CV-A16 infection, then IL-8 was upregulated beginning 12 hours after CV-A16 infection, and finally, TNF-α and IFN-α were also upregulated during the later stage of CV-A16 infection (**Figure 2**; **Supplementary Table 1**). Therefore, these results suggest that CV-A16 infection triggers an inflammatory response.

### IP-10 induced by CV-A16 infection causes the destruction of the BBB

**Figure 3:**
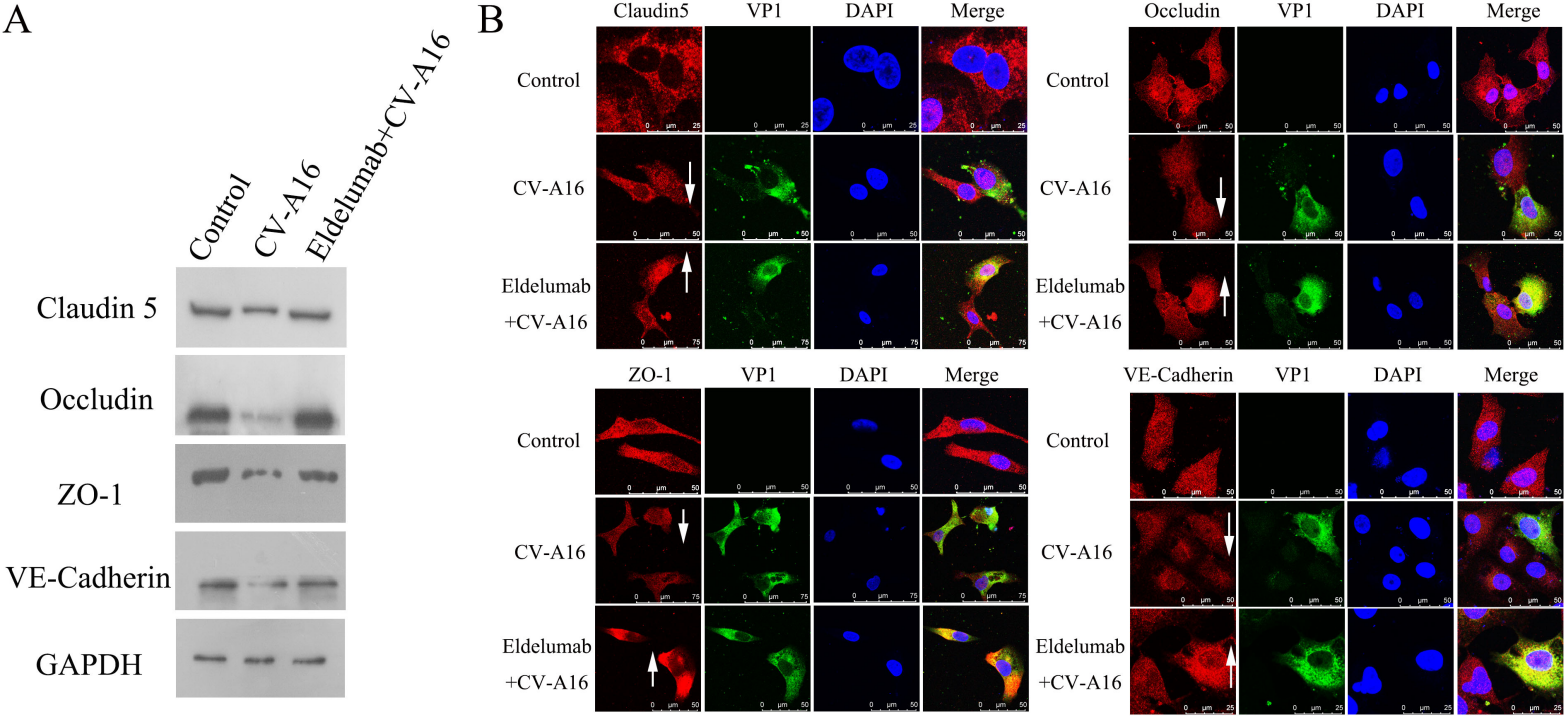
IP-10 is involved in BBB damage. **(A)** WB assay was used to determine the changes in the protein levels of junctional complexes. **(B)** IF staining was used to visually observe changes in the location of junctional complexes.

Accumulating evidence has confirmed that proinflammatory cytokines play crucial roles in BBB dysfunction (28). However, junction complexes between cerebral endothelial cells are essential for maintaining BBB integrity and mainly contain TJ proteins (e.g., Claudin5, Occludin and ZO-1) and AJ proteins (e.g., VE-Cadherin) (29). WB results revealed that CV-A16 infection disrupted the expression of TJ and AJ proteins, but this phenomenon was reversed after the administration of a monoclonal targeting antibody with IP-10 (e.g., Eldelumab) (**Figure 3A**). Subsequently, IF staining further revealed that CV-A16 infection resulted in the downregulation of these proteins, but Eldelumab treatment sharply increased the expression of these proteins in CV-A16-infected cells (**Figure 3B**). Since HUVECs are often used to construct BBB models *in vitro*, combined with our results above, we hypothesized that IP-10 induced by CV-A16 infection may contribute to BBB damage.

### IP-10-induced TNF-α is a key factor in BBB damage

**Figure 4:**
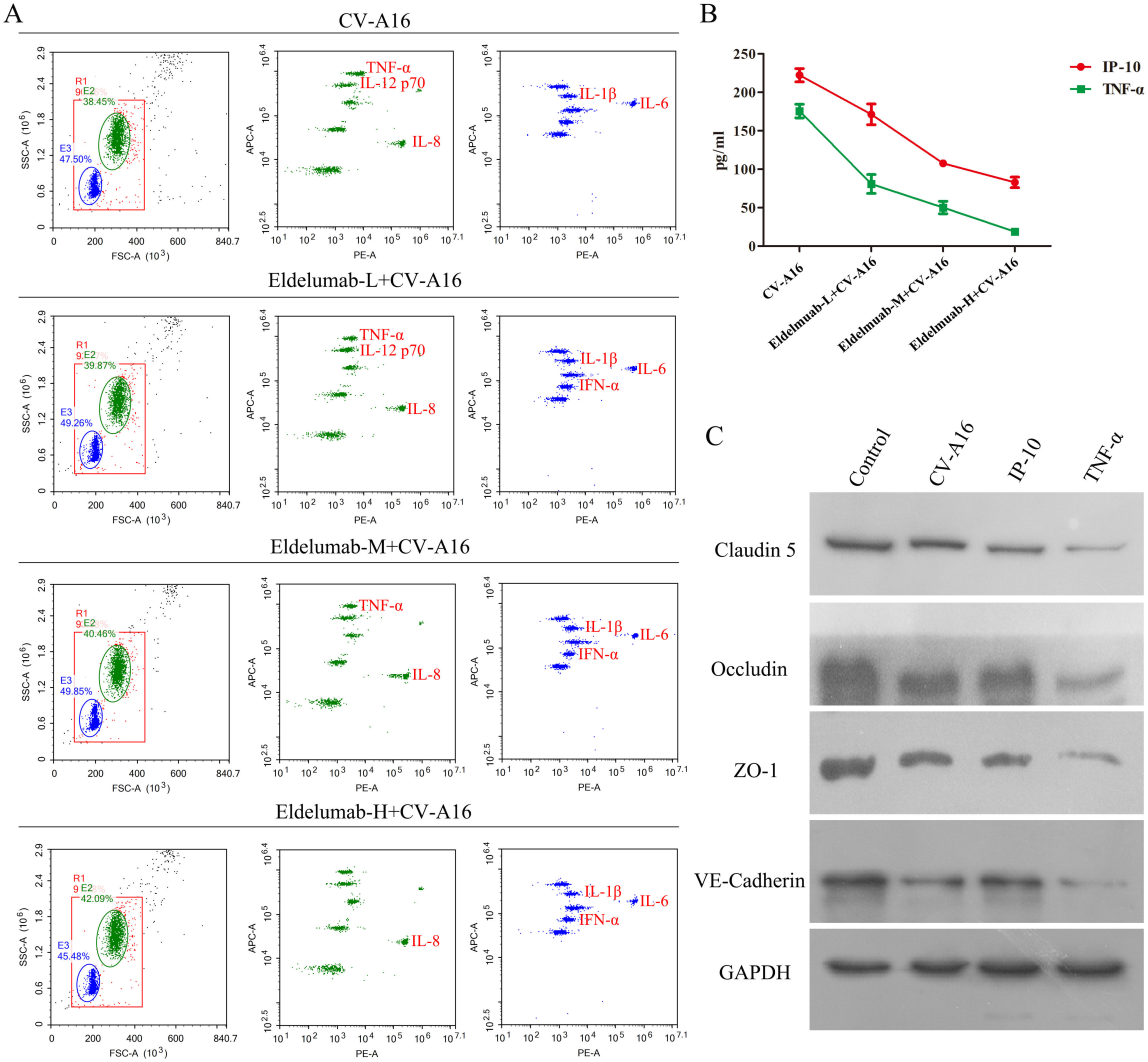
TNF-α contributes to BBB damage. **(A)** Eldelumab was used to clarify the effect of IP-10 on the expression of inflammatory cytokines by flow cytometry in CV-A16-infected cells. **(B)** Eldelumab was used to determine the effect of IP-10 on the expression of TNF-α by ELISA in CV-A16-infected cells. **(C)** Junctional complexes were analyzed with WB after CV-A16 infection, IP-10 treatment and TNF-α treatment. Notes: Eldelumab-L, Eldelumab-M and Eldelumab-H represents different doses of Eldelumab. L indicates a low dose, M indicates a middle dose and H indicates a high dose.

As a chemokine, IP-10 plays an important role in neuroinflammation, primarily by attracting inflammatory cells to cross the BBB, after which it infiltrates the sites in the brain and ultimately promotes the production of chemokines and cytokines, and enhances BBB permeability (24, 30). Therefore, IP-10 is often not the direct cause of BBB damage, but the above results indicate that the inhibition of IP-10 prevents CV-A16-induced BBB damage. Thus, we hypothesized that IP-10 may cause damage to the BBB through its downstream factors. In this study, it was seen that after treatment with Eldelumab, the release of TNF-α from CV-A16-infected cells decreased, but other proinflammatory cytokines, such as IL-6, IL-8, IL-1β and IFN-α, which were enhanced during CV-A16 infection, were not affected by Eldelumab treatment in CV-A16-infected cells (**Figure 4A**). Moreover, it was also discovered that the higher the dose of Eldelumab treatment, the lower the level of TNF-α was released by CV-A16-infected cells, and vice versa (**Supplementary Table 2**). We further tested the concentrations of IP-10 and TNF-α in CV-A16-infected cells treated with different doses of Eldelumab. Our results revealed that the protein release of IP-10 and TNF-α after CV-A16 infection was relatively low when given a high dose of Eldelumab was used, but the protein release of IP-10 and TNF-α after CV-A16 infection was relatively high when given a low dose of Eldelumab was used (**Figure 4B**), which was in line with data of the above flow cytometry data described above. On the basis of these results, we speculated that Eldelumab, as an antibody against IP-10, has a neutralizing effect on the IP-10 protein; thus, in the case of a high dose of Eldelumab, the IP-10 protein released after CV-A16 infection is neutralized by Eldelumab, and TNF-α changes with the amount of IP-10, which further proves that TNF-α may be a downstream molecule of IP-10. To further verify whether TNF-α affects the BBB, we examined the expression of junctional complexes. Our results revealed that, compared with CV-A16 infection and IP-10 treatment, TNF-α treatment markedly reduced the expression of Claudin5, Occludin, ZO-1 and VE-Cadherin. Previous studies have reported that TNF-α, as a cytokine, plays an important role in neuroinflammation mainly by inducing alterations in the organization of junctional complexes that result in BBB dysfunction (31, 32). Thus, our data illustrated that TNF-α may cause BBB damage by disrupting the expression of junctional complexes. Moreover, combined the above results suggest that IP-10 triggered by CV-A16 infection may disrupt the BBB through damage to the BBB junctional complexes caused by its downstream regulation of TNF-α.

### IP-10 and TNF-α lead to BBB damage and accelerate neuroinflammation and viral spread in the mouse brain

**Figure 5:**
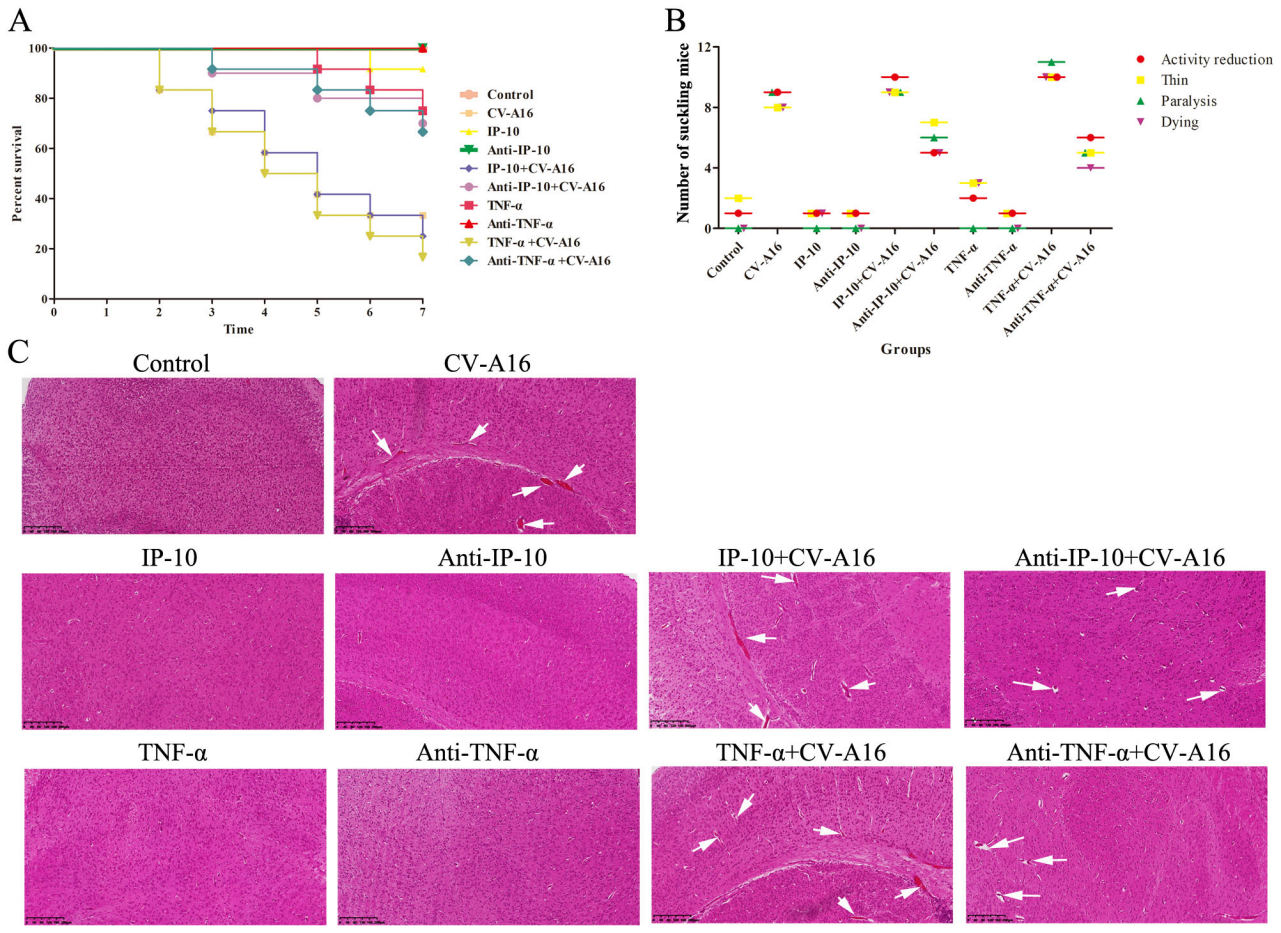
Basic evaluation of suckling mice in each experimental group. **(A)** Survival curves of suckling mice in each group were plotted. **(B)** Clinical symptoms of suckling mice in each group were monitored. **(C)** Pathological changes in the brain tissues were detected by H&E staining and are represented by white arrows.

We next used animal experiments to investigate the associations between IP-10 and TNF-α and their role in the pathological injury of the central nervous system caused by CV-A16 infection, we conducted animal experiments. The TNF-α+CV-A16 group presented the most severe clinical symptoms, the lowest survival rate and the most severe pathological changes in brain tissue (**Figure 5**), but Anti-TNF-α treatment before CV-A16 infection resulted in not only a significant reduction in the clinical symptoms and the degree of pathological injury, but also a significant reduction in the mortality of suckling mice. Although the trends of these indicators in the IP-10+CV-A16 and Anti-IP-10+CV-A16 groups were similar to those in the two groups above, the degree of change in these indicators was significantly lower than that in the two groups above. Therefore, these data suggest that both IP-10 and TNF-α could aggravate the damage caused by CV-A16 infection, and that the effect of TNF-α is more severe. Moreover, compared with IP-10 treatment alone, TNF-α treatment alone resulted in relatively severe clinical symptoms, a relatively lower survival rate and relatively severe pathological changes in brain tissue than IP-10 treatment alone. This result may further confirm our above results shown in **Figure 4**. As it is downstream of IP-10, TNF-α can directly disrupt BBB permeability by destroying intercellular connections; thus, the reason that the relatively more severe effect of TNF-α alone than of IP-10 alone may be because TNF-α acts directly on the BBB, while IP-10 acts indirectly via TNF-α.

**Figure 6:**
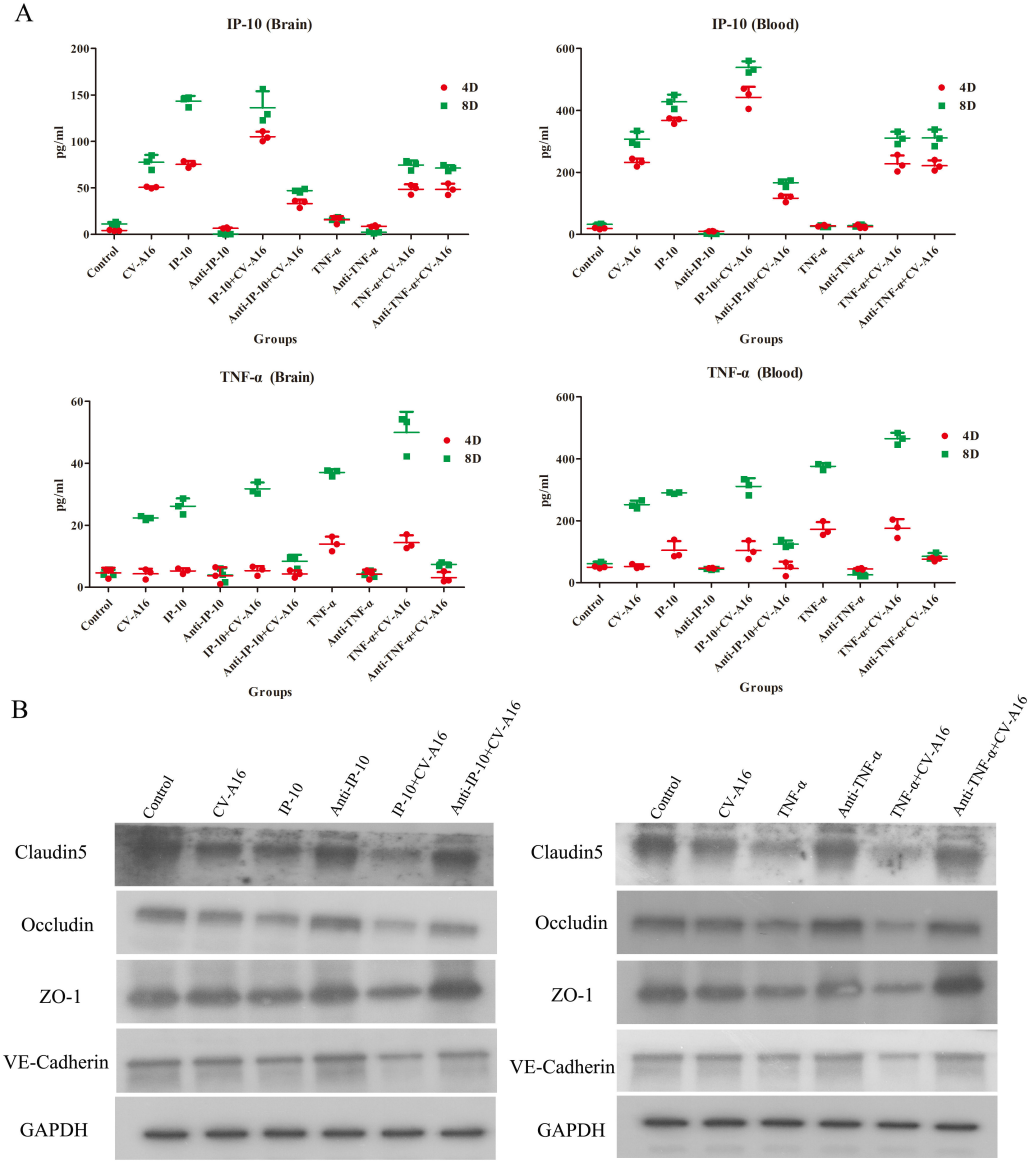
IP-10 and TNF-α aggravate BBB damage caused by CV-A16 infection. **(A)** WB was conducted to evaluate the expression of junctional complexes in brain tissues. **(B)** IHC analysis of brain tissues was performed to determine the expression of junctional complexes.

Next, we measured the concentrations of IP-10 and TNF-α in brain tissues and blood on Day 4 and Day 8 after different treatments. As shown in **Figure 6A**, IP-10 expression was upregulated in the brain tissue and blood of all the groups infected with CV-A16 compared with the control group on both Day 4 and Day 8. Moreover, IP-10 production in the TNF-α+CV-A16 and Anti-TNF-α+CV-A16 groups was not significantly different from that in the CV-A16 group. In addition, there were no differences between the TNF-α/Anti-TNF-α group and the Control group. These results indicate that CV-A16 infection may promote IP-10 production from the beginning of infection and that TNF-α is not associated with IP-10 production. However, TNF-α expression in the brain tissue and blood in the CV-A16 group was not altered on Day 4, but did increase on Day 8. Additionally, TNF-α expression was gradually upregulated in the IP-10, IP-10+CV-A16, TNF-α and TNF-α+CV-A16 groups. These data indicate that CV-A16 infection may increase TNF-α release at later stages of infection and IP-10 induces the release of TNF-α.

Next, the junctional complexes were analyzed. IP-10 treatment had no significant effects on the expression of Claudin5, Occludin, ZO-1 or VE-Cadherin molecules, but TNF-α treatment directly reduced the expression of these molecules (**Figure 6B**). Furthermore, these junctional complexes in the IP-10+CV-A16 and TNF-α+CV-A16 groups were markedly lower than those in the CV-A16 group, but they were markedly greater in the Anti-IP-10+CV-A16 and Anti-TNF-α+CV-A16 groups. Therefore, combined with the above results, we hypothesized that IP-10 induces TNF-α expression, which may further affect BBB integrity.

**Figure 7:**
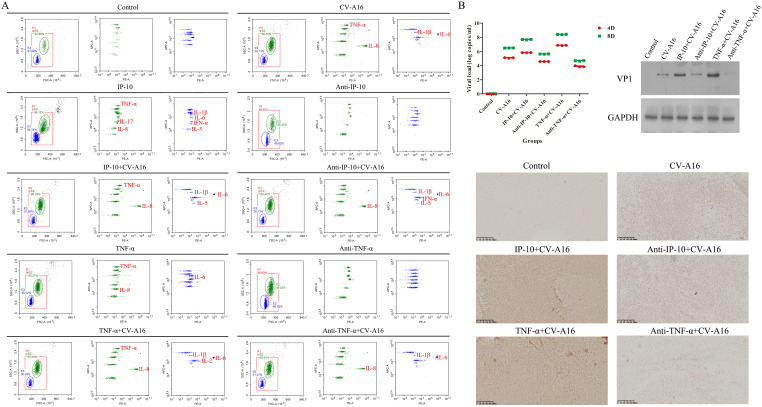
IP-10 and TNF-α exacerbated neuroinflammation and virus spread in the brains of CV-A16-infected mice. **(A)** Inflammatory cytokines in brain tissues were assessed with flow cytometry. **(B)** The virus load, VP1 expression and virus location in brain tissues was determined via qRT-PCR, WB assay and IHC staining, respectively.

Finally, the inflammatory response and CV-A16 loads in the brain tissues were assessed. Flow cytometry revealed that the highest expression of inflammatory cytokines was in the TNF-α+CV-A16 group, followed by the IP-10+CV-A16 group (**Figure 7A**; **Supplementary Table 3**). However, treatment with Anti-TNF-α or Anti-IP-10 dramatically reduced the expression of some inflammatory cytokines, especially TNF-α, in CV-A16-infected cells. In addition, IP-10 treatment alone indeed increased TNF-α expression. These findings suggest that CV-A16 infection causes neuroinflammation, which may be activated by IP-10-induced TNF-α during CV-A16 infection and could be further aggravated via the regulation of TNF-α. On the basis of the results of BBB damage caused by CV-A16 infection, it is possible that CV-A16 is more likely to enter the central nervous system through the damaged BBB. In all groups subjected to CV-A16 infection, IP-10 or TNF-α treatment prominently increased the virus load and VP1 expression in CV-A16-infected cells, whereas Anti-IP-10 or TNF-α treatment noticeably decreased the virus load and VP1 expression in CV-A16-infected cells (**Figure 7B**). Moreover, the viral load and VP1 expression significantly greater in the TNF-α+CV-A16 group than those in the IP-10+CV-A16 group. Therefore, these data suggest that TNF-α and IP-10 may rapidly increase the transmission of CV-A16 in the brain, but the promotion of CV-A16 transmission resulting from IP-10 may be due to its regulation of TNF-α.

## Discussion

EV-A71 and CV-A16 are still the most predominant pathogens for increasingly severe HFMD outbreaks in young children (1). Due to their effects on the CNS, EV-A71 and CV-A16 are considered to constitute a new class of neurotropic viruses after poliovirus (33). Since the inactivated EV-A71 vaccine was developed and put on the market, HFMD caused by EV-A71 has been effectively reduced, but this has also led to a new wave of CV-A16 infection (34, 35). Currently, there are no approved anti-CV-A16 drugs or vaccines available (3), and the mechanism by which the virus gains entry into the CNS and by which CV-A16 stimulates neuroinflammatory lesions remains poorly understood. Thus, in order to prevent and control the spread of CV-A16-induced HFMD, exploring the pathogenesis of CV-A16 infection, especially CNS pathology, is a priority. The BBB, as an important physiological barrier composed of brain microvascular endothelial cells, pericytes, and astrocytes, is the first line of defense that prevents the entry of pathogens from the peripheral circulation into the CNS (36, 37). Many neuroinvasive viruses cause CNS lesions by breaking down the BBB (37, 38). Our previous study had also demonstrated that CV-A16 crosses the BBB and enters the CNS by downregulating miR-1303, which disrupts junctional complexes by directly regulating MMP9 (9). However, if CV-A16 infection is not clinically discerned until after it has already invaded the CNS, the best treatment window for children with CV-A16 infection may be missed. Therefore, this study aimed to identify the key diagnostic molecules and their mechanisms before CV-A16 invades the CNS, which may provide early diagnosis for preventing CV-A16 infection in children from progressing to CNS complications. On the basis of an extensive literature review, we focused on the IP-10 for this purpose (18, 24). IP-10 has reported to be upregulated not only in the serum and CSF of children with HFMD (14), but also frequently in patients with other viral diseases. For example, abnormally high plasma IP-10 levels occur in the context of human immunodeficiency virus (HIV) infection, and IP-10 is considered an important proinflammatory factor in the HIV disease process (20, 39). Hepatitis B virus (HBV) protein X increases IP-10 expression by inducing the activation of NF-κB via the TRAF2/TAK1 signaling pathway, which promotes the migration of leukocytes in response to HBV infection, thus causing pathological immune injury to the liver (40) The early increase in IP-10 in coronavirus disease 2019 (COVID-19) patients not only serves as a potential diagnostic biomarker of COVID-19 (41), but is also closely associated with the severity of COVID-19 disease and the risk of death in COVID-19 patients (42). IP-10 is an important chemokine in the development of airway inflammation caused by influenza virus or respiratory syncytial virus (43). Additionally, IP-10 has been suggested to increase the severity of virus infection and neuronal injury (24, 44), including neurotropic coronavirus (45), tick-borne encephalitis virus infection (46), Zika virus infection (47), and dengue virus infection (48), etc. Therefore, in this study, we first examined the expression of IP-10 in CV-A16-infected HUVECs. Both at the gene level of IP-10 and at the protein level and amount of IP-10 released were clearly elevated during CV-A16 infection. We subsequently evaluated the upstream regulatory pathways that trigger IP-10 expression. Currently, IP-10 induction is thought to be mediated by various signaling pathways, including the JAK-STAT signaling pathway, the NF-κB signaling pathway, and the p38 signaling pathway, etc (18). Among these, we focused on NF-κB because of pivotal role in regulating immune and inflammatory responses (27). Previous studies have shown that innate immunity, as the first line of defense against infectious pathogens, consists of all pattern recognition receptor mediated signaling pathways, such as Toll-like receptors (TLRs), RIG-I-like receptors, Nod-like receptors, and AIM2-like receptors (49, 50). Moreover, these receptors can directly culminate in the activation of the transcription factor NF-κB via the sensing of different molecular signatures of microbial pathogens (51, 52). CV-A16 is a positive-sense single-stranded RNA virus that should be recognized by TLR3, RIG-I, and MDA5 (53). Thus, the proteins of the TLR3-TRIF-TRAF3-TBK1-NF-κB and RIG-I/MDA5-MAVS-TRAF3-TBK1-NF-κB axes were analyzed. Our results revealed that these proteins were gradually upregulated during CV-A16 infection and that NF-κB translocated from the cytoplasm to the nucleus. Normally, NF-κB proteins exist as components of inactive cytoplasmic complexes bound by members of the inhibitor of κB (IκB) family, and once activated, NF-κB is translocated to the nucleus and activates the target genes (26, 54), leading us to hypothesize that the translocation of NF-κB may drive IP-10 gene transcription. To further investigate the above hypothesis, we used NF-κB knockdown and overexpression plasmids. Transfection of the knockdown-NF-κB vector markedly reduced the expression of IP-10 in CV-A16-infected cells; consistently, the overexpression-NF-κB vector markedly elevated the expression of IP-10 in CV-A16-infected cells, which suggested that NF-κB may be a necessary component for the induction of IP-10. As NF-κB is a “master regulator of inflammation”, the expression of inflammatory cytokines was then assessed. As predicted, many inflammatory cytokines, namely, IL-1β, IL-6, IL-8, TNF-α and IFN-α, were notably altered at different time points during CV-A16 infection. Accordingly, in light of combination with the above findings, we hypothesized that CV-A16 infection produces a sequential event, that is, CV-A16 may be recognized by TLR3, RIG-I, and MDA5, which in turn leads to the activation of NF-κB, which further mediates the expression of an array of inflammatory cytokine genes.

Growing evidence has confirmed that peripheral inflammation is crucial for BBB disruption (28). To identify the early inflammatory factors that may destroy the BBB during CV-A16 infection, we observed from the above results that among these inflammatory cytokines, IP-10, IL-1β and IL-6 were already altered in the early stage of CV-A16 infection, whereas TNF-α and IFN-α were altered in the later stage of CV-A16 infection. In fact, IP-10 has been reported to be a predictive marker for immune activation, histopathological damage, or the antiviral response in many virus infections (18), such as hepatitis C virus (55), HIV (56) and Japanese encephalitis virus (57). Therefore, in this study, we first investigated the role of IP-10 in BBB disruption. In line with our previous studies, CV-A16 caused a decrease in the expression of Claudin5, Occludin, ZO-1 and VE-Cadherin, but Eldelumab increased the levels of these proteins in CV-A16-infected HUVECs. These findings suggest that neutralizing IP-10 in CV-A16-infected cells ameliorated the destruction caused by CV-A16 to the BBB. Nevertheless, as a chemokine, the primary role of IP-10 is its chemotactic effect on CXCR3-positive cells, followed by its induction of inflammation (18). Therefore, when considering about the destruction of the BBB caused by IP-10, we hypothesized that the inflammatory effect originating from IP-10 induced damage to BBB integrity, because the greatest threat posed by CV-A16 to children is the inflammatory lesions of the CNS. High, middle and low concentrations of Eldelumab were used to assess the relationship between IP-10 and inflammatory cytokines. Among the 12 inflammatory cytokines, TNF-α was the only cytokine whose expression changed with increasing Eldelumab concentration. ELISAs were subsequently used to determine the concentrations of IP-10 and TNF-α. The results revealed that the IP-10 and TNF-α levels decreased as the dose of Eldelumab increased. Afterward, as determined by WB analysis, the protein levels of junction complexes sharply decreased in HUVECs under TNF-α treatment, followed by IP-10 treatment conditions and finally CV-A16 infection conditions. On the basis of these data, we hypothesized that both IP-10 and TNF-α may cause the destruction of the BBB, but the underlying mechanism may involve TNF-α as a downstream regulatory molecule of IP-10. Moreover, our previous results revealed that IP-10 was upregulated at the initial stage of CV-A16 infection, whereas TNF-α was upregulated at the later stage of CV-A16 infection, which indirectly supports the hypothesis that IP-10 may regulate TNF-α expression. Additionally, the role of TNF-α-induced neuroinflammation in BBB dysfunction has been previously demonstrated in CNS diseases. Therefore, these findings suggest that elevated IP-10 in CV-A16 infection causes BBB destruction, likely by promoting TNF-α expression.

**Figure 8:**
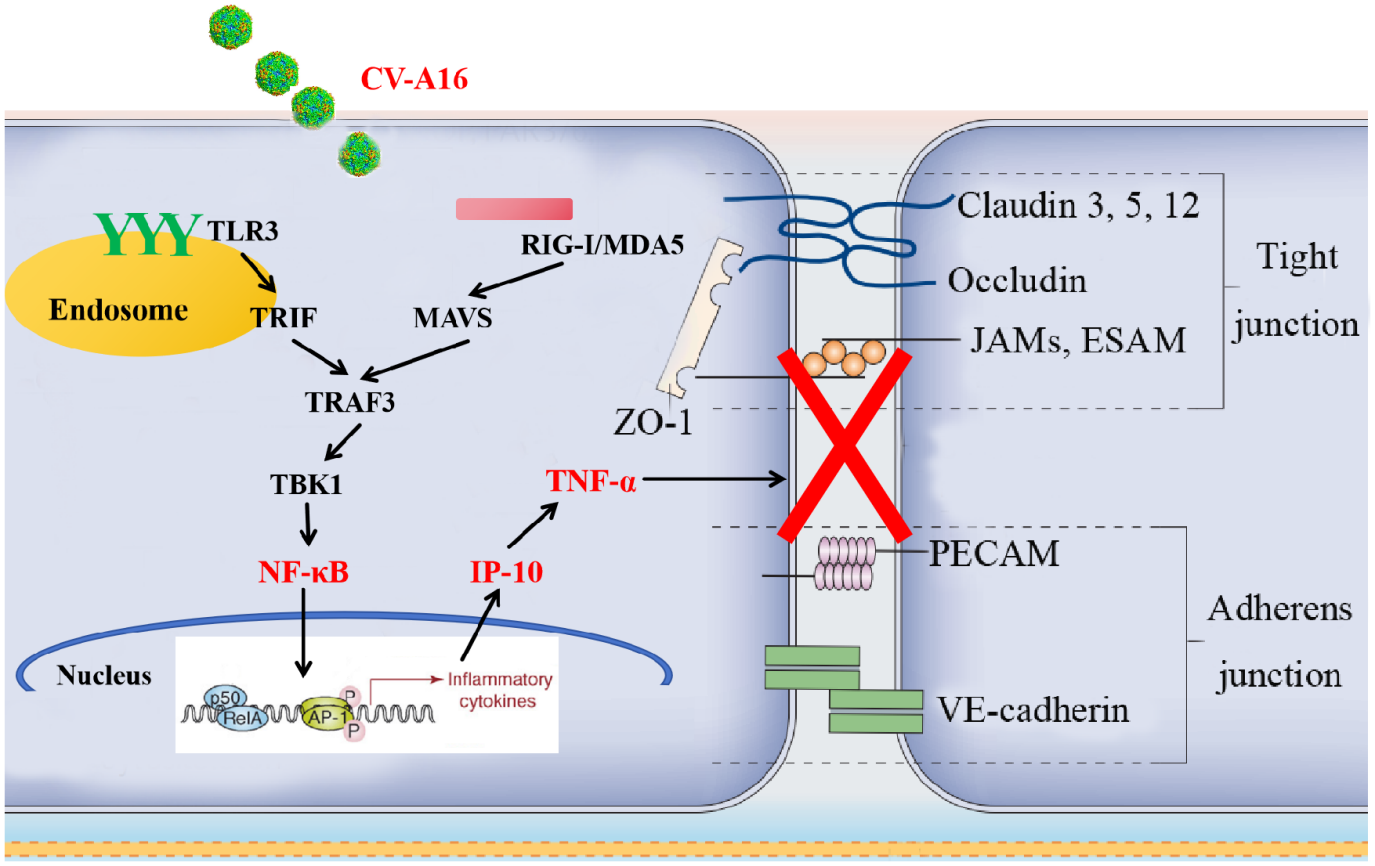
Proposed schematic model of the involvement of IP-10 in the destruction of the BBB.

To further confirm the above conclusions, we utilized a suckling mouse model. Our results first revealed that in the groups of CV-A16-infected mice, the CV-A16-infected mice treated with TNF-α presented the most severe clinical symptoms, the highest fatality rate and the most significant pathological changes, followed by the CV-A16-infected mice treated with IP-10 and finally the CV-A16-infected mice alone. Moreover, these indicators improved in CV-A16-infected mice when the corresponding antibodies (i.e., Anti-TNF-α and Anti-IP-10) were given, which suggests that IP-10 and TNF-α play vital roles in the progression of CV-A16 infection. We subsequently examined the concentrations of IP-10 and TNF-α in brain tissues and blood. Surprisingly, in all the groups treated with IP-10, the expression of TNF-α did not change on Day 4, but was increased on Day 8, and in all the groups treated with Anti-IP-10, the expression of TNF-α also did not change on Day 4 but was weakened on Day 8, indicating that the expression of TNF-α may be regulated by IP-10. Degradation of brain region junctional complexes is a pathological hallmark of BBB destruction caused by neurotropic virus infection (37, 58). In this study, TNF-α treatment not only directly reduced the protein levels of junctional complexes in the mouse brain, but also significantly aggravated decrease in the expression of junctional complexes in CV-A16 infected mice, whereas after Anti-TNF-α treatment, the expression of junctional complexes in CV-A16-infected mice obviously recovered. Although the results of the groups treated with IP-10 were similar to those of the groups treated with TNF-α, the changes in the junctional complexes were not as significant as those in the groups treated with TNF-α. These observations further indicate that TNF-α may be the direct cause of BBB destruction, not IP-10, which instead causes BBB destruction indirectly by promoting TNF-α expression. Indeed, it has been reported that IP-10 promotes BBB destruction by inducing TNF-α during JE virus infection (57), which further supports the regulatory role of the IP-10/TNF-α axis in the BBB in our study. In fact, peripheral inflammation in the form of infection is a common contributing factor for the development and deterioration of CNS diseases, which is mainly due to destruction of the BBB by peripheral inflammation (28). However, peripheral inflammatory cytokines, including TNF-α, IL-1β and IL-6, have also been reported to trigger BBB damage via changes in junctional complexes (59, 60). The results of the analysis of mouse brain inflammatory cytokines not only elucidate the role of IP-10 and TNF-α in neuroinflammation during CV-A16 infection, but also indirectly elucidate the regulatory effect of IP-10 on TNF-α production. Therefore, inflammatory cytokines cause BBB damage by disrupting the intercellular connections at the BBB, which in turn promotes the direct entry of inflammatory cytokines or immune cells into the CNS to induce neuroinflammation, forming a vicious cycle. Finally, we tested the effects of IP-10 and TNF-α on CV-A16 invasion of the CNS. The increased replication of CV-A16 in the IP-10 and TNF-α treatment groups and the decreased replication of CV-A16 in the Anti-IP-10 and Anti-TNF-α treatment groups suggested that elevated CV-A16 in the brain may lead to severe neuroinflammation and neuronal injury. In summary, the evidence presented in this study demonstrate that IP-10 causes BBB breakdown and promotes virus load upregulation in the CNS during CV-A16 infection, primarily by increasing TNF-α expression (**Figure 8**). Moreover, the mechanism by which the IP-10/TNF-α regulatory axis affects BBB integrity is mainly through its destruction of junctional complexes. Additionally, because IP-10 changes early in CV-A16 infection, it serves as a very meaningful early identification factor. Overall, if we give timely treatment by identifying the expression of IP-10 before CNS complications occur in CV-A16-infected children could greatly reduce the occurrence of severe and critical illness and even death in CV-A16-infected children.

## Data Availability

The raw data supporting the conclusions of this article will be made available by the authors, without undue reservation.
