# Supplementary material for: IP-10 acts early in CV-A16 infection to induce BBB destruction and promote virus entry into the CNS by increasing TNF-α expression
Source: Front Immunol. 2024 Nov 4;15:1374447. doi: 10.3389/fimmu.2024.1374447 (PMC11570546; doi:10.3389/fimmu.2024.1374447)
Supplement: Supplementary file 1 [file Table1.docx]

**Table S1.** Identification of inflammatory cytokines at different points during CV-A16 infection.

| Groups | IL-5 | IFN-α | IL-2 | IL-6 | IL-1β | IL-10 | IFN-γ | IL-8 | IL-17 | IL-4 | IL-12 p70 | TNF-α |
| --- | --- | --- | --- | --- | --- | --- | --- | --- | --- | --- | --- | --- |
| CV-A16-0h | 1.33 | <0.83 | 0.21 | 0.44 | 2.94 | <0.46 | 2.84 | 1.56 | <1.27 | <0.46 | 0.6 | 1.14 |
| CV-A16-6h | 0 | 4.07 | 1.01 | **17.73** | **62.65** | 0.32 | 0 | 7.38 | 0.67 | 0.58 | 0.38 | 0.16 |
| CV-A16-12h | 0 | 6.8 | 3.71 | **19.63** | **232.3** | 2.56 | 0.02 | **524.79** | 2.2 | 7.11 | **3.64** | 11.55 |
| CV-A16-24h | 0 | 2.36 | 4.05 | **847.54** | **276.43** | 2.51 | 2.05 | **3268.47** | 2.7 | 6.91 | 1.8 | 13.43 |
| CV-A16-48h | 0 | **9.95** | 5.13 | **2943.75** | **312.66** | 2.73 | 0 | **3297.08** | 2.59 | 7.56 | 0.86 | **30.91** |
| CV-A16-72h | 0 | **11.64** | 5.49 | **3830.22** | **316.3** | 2.73 | 3.87 | **3180.76** | 2.62 | 8.05 | 2.87 | **175.51** |

**Table S2.** Identification of inflammatory cytokines in CV-A16-infected HUVECs subjected to different treatments. Notes: Eldelumab-L, Eldelumab-M and Eldelumab-H indicate low, middle and doses of Eldelumab, respectively.

| Groups | IL-5 | IFN-α | IL-2 | IL-6 | IL-1β | IL-10 | IFN-γ | IL-8 | IL-17 | IL-4 | IL-12 p70 | TNF-α |
| --- | --- | --- | --- | --- | --- | --- | --- | --- | --- | --- | --- | --- |
| CV-A16 | 0 | 8.2 | 5.19 | **3760** | **313.31** | 2.93 | 0.56 | **3272.87** | 2.81 | 7.92 | **3.55** | **167.55** |
| Eldelumab-L+CV-A16 | 0 | **10.44** | 6.2 | **4369** | **301.25** | 2.88 | 3.77 | **3180.98** | 3.54 | 8.42 | **4.35** | **61.16** |
| Eldelumab-M+CV-A16 | 0 | **8.99** | 6.17 | **4001.59** | **326.18** | 2.73 | 0.19 | **3738.81** | 2.46 | 7.86 | 3.33 | **46.79** |
| Eldelumab-H+CV-A16 | 0 | **14.04** | 6.14 | **3776.15** | **341.97** | 2.78 | 1.85 | **3547.24** | 2.78 | 8.3 | 2.33 | 15.76 |

**Table S3.** Identification of inflammatory cytokines in the brain tissues of mice.

| Groups | IL-5 | IFN-α | IL-2 | IL-6 | IL-1β | IL-10 | IFN-γ | IL-8 | IL-17 | IL-4 | IL-12 p70 | TNF-α |
| --- | --- | --- | --- | --- | --- | --- | --- | --- | --- | --- | --- | --- |
| Control | 1.33 | 1.63 | 1.68 | 1.65 | 1.97 | 1.08 | 0.82 | 7.25 | 2.92 | 1.56 | 1.91 | 1.02 |
| CV-A16 | 5.84 | 6.26 | 3.16 | **5657.29** | **31.51** | 0.36 | 2.59 | **5699.3** | 0.81 | 0.81 | 0.8 | **22.4** |
| IP-10 | **27.33** | **9.12** | 6.55 | **8.29** | **27.2** | 6.45 | 3.29 | **21.62** | **25.5** | 0.78 | 4.91 | **26.21** |
| Anti-IP-10 | 0.11 | 1.36 | 1.61 | 4.7 | 0.31 | 2.32 | 3.52 | 1.64 | 9.08 | 1.35 | 1.36 | 2.34 |
| IP-10+CV-A16 | **11.1** | 7.18 | 6.71 | **15741.35** | **86.99** | 0.51 | 6.36 | **7199.37** | 1.01 | 0.68 | 0.94 | **31.37** |
| Anti-IP-10+CV-A16 | **27.99** | **8.92** | 5.85 | **8577.49** | **37.67** | 0.34 | 2.96 | **6497.72** | 0.72 | 0.69 | 1.01 | 7.82 |
| TNF-α | 6.42 | 4.1 | 2.85 | **29.54** | 6.59 | 3.15 | 13.97 | **66.96** | 6.07 | 3.84 | 5.17 | **31.96** |
| Anti-TNF-α | 0.79 | 2.25 | 2.56 | 1.43 | 0 | 1.68 | 3.89 | 1.78 | 9.63 | 1.36 | 1.44 | 1.88 |
| TNF-α+CV-A16 | 0 | 2.88 | **9.06** | **19781.64** | **52.64** | 0.4 | 3.56 | **7619.78** | 1.03 | 0.69 | 1.24 | **47.78** |
| Anti-TNF-α+CV-A16 | 0.33 | 8.25 | 4.24 | **8004.55** | **87.27** | 0.37 | 2.18 | **5410.03** | 0.58 | 0.66 | 0.81 | 7.75 |
